# Do problematic gamblers and loot boxers share similar fallacies of thought? A comparative analysis of cognitive biases

**DOI:** 10.3389/fpsyg.2024.1430926

**Published:** 2024-11-26

**Authors:** Francisco J. Sanmartín, Judith Velasco, Mario Gálvez-Lara, Fátima Cuadrado, Juan A. Moriana

**Affiliations:** ^1^Department of Psychology, University of Cordoba, Cordoba, Spain; ^2^Maimonides Institute for Biomedical Research of Cordoba (IMIBIC), Cordoba, Spain; ^3^Reina Sofia University Hospital, Cordoba, Spain

**Keywords:** loot boxes, gambling, video games, gambling-related cognitions, cognitive bias, behavioural addictions

## Abstract

Cognitive biases are associated with the beginning and maintenance of addictive behaviours. While these biases have been studied in gambling, they have yet to be thoroughly investigated in the context of loot boxes (LBs), largely because of the relatively recent emergence of this phenomenon. This study compared cognitive biases in problematic gamblers, non-problematic gamblers, LB purchasers, and free-LB openers. For this aim, 279 participants (63.1% males) with a mean age of 23.65 years (*SD* = 8.66) completed a self-report. The results showed no differences between problematic gamblers, LB purchasers and LB openers on illusion of control and predictive control. In contrast to LB openers, problematic gamblers and LB purchasers obtained statistically similar scores on interpretative biases, gambling-related expectancies and the total score of the Gambling Related Cognitions Scale (GRCS). Only problematic gamblers experienced a higher perceived inability to stop gambling. Moreover, problematic gamblers, LB purchasers and LB openers scored higher on all biases compared to non-problematic gamblers. Eighty-six participants simultaneously gambled and used LBs. When this overlap was controlled, problematic gamblers and loot boxers shared all cognitive biases but the perceived inability to stop gambling; and scored statistically higher than non-problematic gamblers in all cognitive biases except for the illusion of control. The study provides additional evidence of the relationship between gambling and LBs.

## 1 Introduction

Video games have become one of the main digital entertainment options worldwide (Hodent, 2020), with approximately 3.1 billion players (DFC Intelligence, 2020). Previous studies have found evidence suggesting that video game use has positive effects (e.g., improved attentional and spatial skills, positive emotions, socialisation; Granic et al., 2014). However, over the past few years, growing attention has been given to a negative aspect that is becoming increasingly common: gambling-related dynamics incorporated as a method of monetisation within video games. One of the systems that has attracted most interest is loot boxes.

Loot boxes (LBs) are virtual crates that appear in video games. They can be accessed through different ways, either for free (i.e., after achieving a concrete goal during the gameplay) or by purchasing using real or virtual currency to receive random objects (Zendle et al., 2019). It is possible to differentiate between players who only open LBs for free (free-LB openers) and players that, in addition to opening free LBs, pay for them to increase the probability of obtaining the items they want (LB purchasers). Although most studies have treated both types of players as a single group, certain aspects may differentiate them. LB purchasers present higher gambling expenditure and frequency, gambling-related problems, and gambling harms (e.g., debt accumulation, distress; Rockloff et al., 2021; Russell et al., 2023) compared to free-LB openers, which suggests greater gambling engagement.

Previous research has associated LBs with gambling, finding a positive relationship between gambling severity and the use of LBs (Garea et al., 2021; González-Cabrera et al., 2024). This relationship, which has led LBs to be considered a form of gambling, appears to be based on the existence of common features between the two dynamics; namely, prepayment, random rewards, the possibility of earning real money through sales, intermittent reinforcement (Drummond et al., 2020), near misses (i.e., a losing situation that closely approximates a win without achieving it), and powerful auditory and visual stimuli (i.e., lights, colours, and sounds; Derevensky and Griffiths, 2019). These characteristics may partially explain the addictive potential of LBs (understood as the beginning and maintenance of consumption). Cognitive biases may be a contributing variable to this association (Barrault and Varescon, 2013; Ciccarelli et al., 2016) although they have not been extensively investigated in LBs.

Cognitive biases are defined as ‘systematic (that is, non-random and, thus, predictable) deviation from rationality in judgment or decision-making’ (Blanco, 2017). Kahneman (2011) argues that humans process information through two systems: (a) system 1, which operates fast and automatically and involves minimum effort, and (b) system 2, which operates slowly and deliberately and requires greater exertions. When a stimulus draws our attention, system 1 responds first and, if the response requires minimal effort, it does not mobilise system 2. In this circumstance, cognitive biases would arise, as system 1 operates automatically through heuristics (i.e., mental cutoffs), while system 2 does not perceive cues of an error. Alternatively, when it detects such errors, system 2 approves the intuitive response of system 1 without monitorisation, that is, without further checking.

Several cognitive biases have been described for gambling. As there is no consensus on their denomination or characterisation (Labrador and Labrador, 2021), the same bias can be found with different names (Goodie et al., 2019), which has contributed to conceptual difficulties in this research field. Among the most studied biases are illusion of control (Dixon et al., 2018), gambler’s fallacy (Leonard et al., 2021), near-misses (Barton et al., 2017), and interpretative biases (Ledgerwood et al., 2020). Some of these biases have been associated with heuristics such as availability (i.e., illusory correlations, inherent memory bias, and the availability of others’ wins), and representativeness (i.e., the gambler’s fallacy, overconfidence, and number picking trends). Other biases, however, such as illusion of control, near-miss effects, self-serving bias (i.e., attribution of wins to skill and losses to external factors), and the concept of impaired control, have not been linked to any particular heuristic (Goodie and Fortune, 2013). Check Jacobsen et al. (2007) for an extensive analysis of cognitive biases in gambling.

Previous studies have examined differences in cognitive biases in problematic gamblers, finding that they tend to present higher scores on illusion of control, predictive control (also known as gambler’s fallacy), interpretative biases, gambling-related expectancies, and the perceived inability to stop gambling (deteriorated control; Oei et al., 2008; Tang and Wu, 2012; Cosenza et al., 2014; Ciccarelli et al., 2017; Tani et al., 2018), along with greater illusory correlation (Labrador et al., 2020) and belief in luck and perseverance (Orlowski et al., 2020) than non-problematic gamblers.

Considering the suggested convergence between gambling and LBs, it is possible to hypothesise the existence of other common characteristics between gamblers, LB purchasers and free-LB openers, including shared cognitive biases. However, to the best of the author’s knowledge, only one study to date has explored this relationship, finding a positive association between cognitive distortions and risky LB use (Brooks and Clark, 2019). Thus, the present study aims to examine the presence of shared cognitive biases among problematic gamblers, LB purchasers and free-LB openers. It is expected that problematic gamblers, LB purchasers, and free-LB openers will have similar levels of each cognitive bias and that they will be greater than the biases of the non-problematic gamblers group.

## 2 Methods

### 2.1 Participants

The sample comprised 279 participants aged 18 to 68 years old (*M* = 23.65, *SD* = 8.66). The gender distribution was not equally represented [*χ*^2^ (1, *N* = 279) = 19.100; *p* < 0.001], with 176 males (63.1%) and 103 females (36.9%). Participants were divided into four groups: (a) problematic gamblers (*n* = 41), consisting of individuals who received psychological treatment for gambling-related problems in specialised institutions but had gambled in the past year (i.e., they had relapsed), and who also obtained a score equal to or higher than eight on the Problem Gambling Severity Index (PGSI); (b) LB purchasers (*n* = 64); (c) free-LB openers (*n* = 104); and (d) non-problematic gamblers (*n* = 70) composed of individuals who had never opened or purchased LBs but who stated they had gambled in the past 12 months and who scored lower than eight on the PGSI. In the case of LBs users, if they had gambled and scored lower than eight on the PGSI, they were classified into their respective group (LB purchaser or free-LB opener); however, if they scored eight or higher on the PGSI, they were excluded from the study as they were considered problematic gamblers but had not received psychological treatment. Sociodemographic data are shown in Table 1.

**Table 1:** Sociodemographic data.

		Problematic gamblers	LB purchasers	LB openers	Non-problematic gamblers
		*M (SD)*	*M (SD)*	*M (SD)*	*M (SD)*
Age		34.71 (13.18)	22.89 (6.13)	22.15 (6.20)	20.07 (4.53)
		*n* (%)	*n* (%)	*n* (%)	*n* (%)
Sex	Male	37 (90.2)	60 (93.8)	74 (71.2)	5 (7.1)
	Female	4 (9.8)	4 (6.3)	30 (28.8)	65 (92.9)
Status	Single	30 (73.2)	56 (91.8)	95 (96)	68 (97.1)
	Married	10 (24.4)	3 (4.9)	3 (3)	2 (2.9)
	Divorced/Separated	1 (2.4)	-	1 (1)	-
	Widowed	-	2 (3.3)	-	-
Highest academic level completed	Primary Education	5 (12.2)	1 (1.6)	1 (1)	-
	Secondary Education	7 (17.1)	8 (13.1)	8 (8.1)	-
	GCE	15 (36.6)	32 (52.5)	57 (57.6)	47 (67.1)
	VET	7 (17.1)	13 (21.3)	22 (22.2)	23 (32.9)
	University Degree	5 (12.2)	5 (8.2)	10 (10.1)	-
	Postgraduate (Masters)	2 (4.9)	2 (3.3)	1 (1)	-
Currently employed	Yes	24 (58.5)	25 (41)	19 (19.2)	8 (11.4)
	No	17 (41.5)	36 (59)	80 (80.8)	62 (88.6)
Socioeconomic status	Low	9 (22)	-	3 (3)	1 (1.4)
	Medium-low	8 (19.5)	13 (21.3)	18 (18.2)	16 (22.9)
	Medium	21 (51.2)	37 (60.7)	61 (61.6)	46 (65.7)
	Medium-high	2 (4.9)	11 (18)	15 (15.2)	6 (8.6)
	High	1 (2.4)	-	2 (2)	1 (1.4)

GCE, general certificate of education; VET, vocational education and training.

### 2.2 Instruments

Ad hoc questionnaire: for this study, a survey was specifically designed on sociodemographic data, LB use (i.e., opening and purchasing, frequency of LB use), and gambling habits (i.e., gambling behaviour and gambling frequency).

Problem Gambling Severity Index (PGSI; Ferris and Wynne, 2001): Spanish validation by López-González et al. (2018). This instrument assesses the severity of gambling using nine items distributed on a 4-point scale (0 = never; 1 = sometimes; 2 = most of the time; and 3 = almost always). Scores range from 0 to 27 points and classify individuals into four groups (0 = non-problem gambling, 1–2 = low-risk gambling; 3–7 = moderate-risk gambling; and 8 or more = problem-gambling). The internal consistency of the PGSI is 0.84 in the original version and 0.97 in the Spanish version.

Gambling Related Cognitions Scale (GRCS; Raylu and Oei, 2004): Spanish validation by del Prete et al. (2017). The GRCS includes 23 items that assess cognitions related to gambling on five subscales: illusion of control (e.g., ‘I have specific rituals and behaviours that increase my chances of winning’), predictive control (e.g., ‘I have some control over predicting my gambling wins’), interpretative biases (e.g., ‘Remembering how much money I won last time makes me continue gambling’), gambling-related expectancies (e.g., ‘Gambling makes things seem better’) and the perceived inability to stop gambling (e.g., ‘I’m not strong enough to stop gambling’). Items are distributed on a 7-point Likert scale (1 = strongly disagree; 2 = moderately disagree; 3 = mildly disagree; 4 = neither agree nor disagree; 5 = mildly agree; 6 = moderately agree; 7 = strongly agree). Given that the GRCS does not provide cut-off scores, higher scores indicate greater distorted cognitions related to gambling. Total scores range from 23 to 161 points. The scale has an internal consistency of 0.93 in the original version and 0.95 in the Spanish version.

Adaptation of the Gambling-Related Cognitions Scale to Loot Box use (GRCS-LB): to evaluate distorted cognitions in openers and purchasers of LBs, GRCS was adapted to LB users. For this aim, terms related to gambling were reworded with respect to LB consumption (see Supplementary material), following the strategy of previously published studies (Nolen, 1988; Jeong and Oh, 2020; Chae, 2022; Lambert et al., 2022). The internal consistency of the adapted GRCS was 0.90.

### 2.3 Procedure

To compose the sample of problematic gamblers, a search was carried out of all Spanish institutions dedicated to the treatment of gambling, resulting in the identification of 64 institutions that were approached by email. The response rate was 15.6% (*n* = 10). Two months later, associations were contacted again to increase participation; however, only those associations that agreed to participated in the study in the first call responded.

For the sample of LB purchasers and free-LB openers, a search for all Spanish competitive e-sports teams (i.e., FIFA, Brawl Stars, Clash Royale, League of Legends, and Rocket League) was undertaken. A total of 424 teams were identified and approached both by email and Twitter. In this case, 1.2% (*n* = 5) responded to the email and 7.5% (*n* = 32) to the private Twitter messages. To broaden participation among this group, a competitive e-sports community (WeClutch) was contacted. The response rate was 20.6% (*n* = 36). Finally, the non-problematic gamblers group included undergraduate students from the University of Cordoba, Spain.

Participants interested in collaborating with the study received a Google form containing a statement about the purpose of the project along with the eligibility conditions (being over 18 years old) and the estimated duration of the survey. Data anonymity and confidentiality were ensured. Participation was voluntary and the survey required 10–15 min to complete. As an incentive, participants were given the option of entering a prize draw to win a gift card for different platforms (e.g., Spotify, FIFA points, DAZN).

Participants completed slightly different versions of the questionnaire based on their reported gambling and LB behaviours. Concretely, participants who reported engaging in only one of these activities (either gambling or LB use) completed the GRCS version to that condition: those who gambled but did not use LBs completed only the GRCS, while those who used LBs, but did not gamble responded only to the GRCS-LB. Participants who reported both gambling and LB use completed both the GRCS and the GRCS-LB. Finally, participants who reported neither using LBs nor gambling were excluded from the study. Additionally, participants who reported gambling completed the PGSI to assess the severity of their gambling behaviour.

### 2.4 Data analysis

Prior to data analysis, the data were checked for low quality or invalid responses (e.g., acquiescence, missing-values, outliers), by analysing response patterns. No response needed to be eliminated. Normality assumptions were tested for the dependent variables (illusion of control, predictive control, interpretative biases, gambling-related expectancies, perceived inability to stop gambling, and total GRCS score), with a *p*-value <0.05 (Kolmogorov–Smirnov). Therefore, nonparametric tests were performed. For the comparison of means between several independent samples (problematic gamblers, LB purchasers, LB openers, and non-problematic gamblers), Kruskal–Wallis *H* tests were performed. *Post hoc* comparisons were performed using the Mann–Whitney *U* test.

Each test is accompanied by its effect size, which is the squared epsilon statistic (*ε*^2^_R_) for the Kruskal–Wallis *H* test and Pearson’s correlation coefficient (*r*) for the Mann–Whitney *U* test (Tomczak and Tomczak, 2014). Data analysis was performed with the IBM SPSS Statistics version 25 statistical package assuming a significance level of *p* = 0.05.

## 3 Results

### 3.1 Characterisation of the sample

#### 3.1.1 Characterisation of problematic gamblers sample

Of the sample of problematic gamblers (*N* = 41), 46.3% reported having played video games. Of problematic gamblers who played video games, 68.4% reported having opened LBs in the past 3 months. Of these, 30.8% opened less than 20 LBs, 61.5% opened 20–50 LBs, and 7.7% opened more than 100 LBs. In relation to purchases, 26.3% indicated that they had bought LBs. Forty percent bought 1 to 5 LBs; 40% bought 6 to 10; and 20% bought 11 to 15.

Regarding the frequency of gambling, 34.1% of problematic gamblers reported having gambled daily in the past 12 months, about 22% said they had played weekly, and 17% had played monthly. Of the remaining respondents, 17% stated that they had gambled at least once in the past 6 months or year. Of the total participants, 9.8% did not indicate gambling frequency, although they responded that they had gambled in the previous year.

#### 3.1.2 Characterisation of loot box openers

The sample included 168 loot boxers (purchasers and openers). Of these, 61.9% stated they had only opened free LBs in the past 3 months. Among the openers, 65.4% reported having opened less than 20 boxes, 17.3% had opened 20 to 50, 8.7% had opened 50 to 100, and 8.7% more than 100 LBs.

A total of 39.4% of the openers reported having gambled over the past year, of which 9.6% reported gambling weekly, 9.6% monthly, 8.7% semi-annually, and 11.5% annually. A total of 60.6% of the respondents indicated that they had not gambled in the past year.

#### 3.1.3 Characterisation of the loot box purchasers

Among LB consumers (*N* = 168), 38.1% had bought at least one box in the past 3 months. Of those who purchased LBs, 62.5% acquired 1 to 5 LBs, 18.8% bought 6 to 10, 4.7% purchased 11 to 15, 3.1% bought 16 to 20, and 10.9% acquired more than 25.

Concerning gambling behaviour, half of the sample reported having gambled in the past year. Within this period, 6.3% gambled daily, 4.7% weekly, 15.6% once a month, 10.9% once every 6 months, and 12.5% once a year.

#### 3.1.4 Characterisation of the non-problematic gamblers group

None of the participants (*N* = 70) had ever played video games, therefore they had never opened or purchased loot boxes. In contrast, all participants had gambled in the past year. Among the non-problematic gamblers, 4.3% reported having gambled weekly, 22.9% monthly, 40% once every six months, and 32.9% once a year.

### 3.2 Comparison of cognitive biases between problematic gamblers, LB purchasers, free-LB openers, and the non-problematic gamblers group

Statistically significant differences were found between the groups for each cognitive bias and for the total GRCS score: illusion of control [*χ*^2^ (3) = 30.36; *p* < 0.001; *ε^2^_R_* = 0.11], predictive control [*χ*^2^ (3) = 42.15; *p* < 0.001; *ε^2^_R_* = 0.16], interpretative biases [*χ*^2^ (3) = 71.55; *p* < 0.001; *ε^2^_R_* = 0.26], gambling-related expectancies [*χ*^2^ (3) = 104.80; *p* < 0.001; *ε^2^_R_* = 0.38], perceived inability to stop gambling [*χ*^2^ (3) = 73.56; *p* < 0.001; *ε^2^_R_* = 0.26], and total GRCS score [*χ*^2^ (3) = 85.36; *p* < 0.001; *ε^2^_R_* = 0.31]. Group means, standard deviations, and *post hoc* comparisons are shown in Tables 2, 3.

**Table 2:** Means and standard deviations on the gambling related cognitions scale (GRCS).

Cognitive bias	Before controlling the overlap between the groups				After controlling the overlap between the groups		
	Problematic gamblers (*N* = 41)	LB purchasers (*N* = 64)	LB openers (*N* = 104)	Non-problematic gamblers (*N* = 70)	Problematic gamblers (*N* = 28)	Loot Boxers (*N* = 95)	Non-problematic gamblers (*N* = 70)
	*M (SD)*	*M (SD)*	*M (SD)*	*M (SD)*	*M (SD)*	*M (SD)*	*M (SD)*
Illusion of control	9.32 (6.05)	9.80 (5.50)	8.75 (4.21)	5.86 (3.05)	7.71 (4.89)	9.16 (4.75)	5.86 (3.05)
Predictive control	17.20 (9.90)	16.08 (7.90)	16.07 (6.27)	10.11 (5.35)	15 (8.53)	15.49 (6.40)	10.11 (5.35)
Interpretative biases	14.46 (6.72)	12.03 (5.65)	11.88 (5.78)	5.94 (3.20)	13.25 (6.67)	11.11 (5.19)	5.94 (3.20)
Gambling-related expectancies	14.51 (6.84)	12.61 (4.85)	11.70 (4.73)	5.41 (2.28)	12 (6.21)	11.84 (4.22)	5.41 (2.28)
Perceived inability to stop gambling	17.66 (9.53)	9.55 (5.92)	9.31 (5.33)	5.64 (1.32)	15.96 (9.17)	8.81 (4.55)	5.64 (1.32)
Total GRCS score	73.15 (33.45)	60.06 (26.10)	57.71 (20.95)	32.97 (12.84)	63.93 (29.95)	56.41 (20.02)	32.97 (12.84)

Participants classified as gamblers completed the GRCS, while participants classified as loot box users completed the GRCS-LB.

**Table 3:** Post hoc comparisons on the gambling related cognitions scale (GRCS).

	Comparisons		*U*	*p*	*r*
Illusion of control	Problematic gamblers	LB purchasers	1,207	0.485	0.07
		LB openers	2,109	0.919	0.01
		Non-problematic gamblers^*^	967	0.003^*^	0.29
	LB purchasers	LB openers	3,076	0.406	0.06
		Non-problematic gamblers^*^	1168.5	<0.001^*^	0.42
	LB openers	Non-problematic gamblers^*^	2068.5	<0.001^*^	0.37
Predictive control	Problematic gamblers	LB purchasers	1,293	0.900	0.01
		LB openers	2110.5	0.925	0.01
		Non-problematic gamblers^*^	841.5	<0.001^*^	0.35
	LB purchasers	LB openers	3,179	0.626	0.04
		Non-problematic gamblers^*^	1,123	<0.001^*^	0.43
	LB openers	Non-problematic gamblers^*^	1,584	<0.001^*^	0.48
Interpretative biases	Problematic gamblers	LB purchasers	1028.5	0.062	0.18
		LB openers^*^	1638.5	0.03^*^	0.18
		Non-problematic gamblers^*^	414	<0.001^*^	0.61
	LB purchasers	LB openers	3,260	0.824	0.02
		Non-problematic gamblers^*^	770.5	<0.001^*^	0.58
	LB openers	Non-problematic gamblers^*^	1,347	<0.001^*^	0.54
Gambling-related expectancies	Problematic gamblers	LB purchasers	1101.5	0.166	0.14
		LB openers^*^	1,614	0.023^*^	0.19
		Non-problematic gamblers^*^	331.5	<0.001^*^	0.66
	LB purchasers	LB openers	2923.5	0.185	0.10
		Non-problematic gamblers^*^	345	<0.001^*^	0.74
	LB openers	Non-problematic gamblers^*^	777.5	<0.001^*^	0.67
Perceived inability to stop gambling	Problematic gamblers	LB purchasers^*^	661.5	<0.001^*^	0.42
		LB openers^*^	1001.5	<0.001^*^	0.42
		Non-problematic gamblers^*^	292.5	<0.001^*^	0.71
	LB purchasers	LB openers	3162.5	0.581	0.04
		Non-problematic gamblers^*^	1,034	<0.001^*^	0.5
	LB openers	Non-problematic gamblers^*^	1984	<0.001^*^	0.41
Total GRCS score	Problematic gamblers	LB purchasers	1027.5	0.062	0.18
		LB openers^*^	1601.5	0.020^*^	0.19
		Non-problematic gamblers^*^	333	<0.001^*^	0.64
	LB purchasers	LB openers	3326.5	0.996	0.001
		Non-problematic gamblers^*^	627	<0.001^*^	0.62
	LB openers	Non-problematic gamblers^*^	1074.5	<0.001^*^	0.60

* Statistical significance *p* ≤ 0.05.

Participants classified as gamblers completed the GRCS, while participants classified as loot box users completed the GRCS-LB.

Results from the *post hoc* tests indicated that problematic gamblers and LB purchasers did not have statistically significant differences in several cognitive biases (i.e., illusion of control, predictive control, interpretative biases, and gambling-related expectancies). Likewise, problematic gamblers and LB openers obtained statistically similar scores in illusion of control and predictive control, but not in the other cognitive biases.

Problematic gamblers, LB purchasers and free-LB openers presented statistically significant differences when compared to the non-problematic gamblers group, both in the cognitive biases and in the total GRCS score. Problematic gamblers, LB purchasers and free-LB openers scored higher than the non-problematic gamblers group.

Finally, no statistically significant differences were observed between LB purchasers and LB openers in any of the cognitive biases assessed or in the total GRCS score.

### 3.3 Cognitive biases comparison after controlling the overlap between the groups

Because some problematic gamblers used LBs, LB purchasers also opened free-LB, and some LB users had gambled past year, the sample was adjusted to examine the impact of a potential overlap between the groups. Thirteen participants in the group of problematic gamblers who used LBs were excluded from the sample, leaving 28 individuals. The groups of LB openers and LB purchasers were merged into a group named “loot boxers” which was initially composed of 168 participants. After removing LB users who also gambled last year (*n* = 73), the loot boxers comprised 95 participants who only consumed LBs.

Statistically significant differences were found between the groups for each cognitive bias and for the total GRCS score: illusion of control [*χ*^2^ (2) = 23.97; *p* < 0.001; *ε^2^_R_* = 0.12], predictive control [*χ*^2^ (2) = 31.31; *p* < 0.001; *ε^2^_R_* = 0.16], interpretative biases [*χ*^2^ (2) = 52.86; *p* < 0.001; *ε^2^_R_* = 0.28], gambling-related expectancies [*χ*^2^ (2) = 85.61; *p* < 0.001; *ε^2^_R_* = 0.45], perceived inability to stop gambling [*χ*^2^ (2) = 56.71; *p* < 0.001; *ε^2^_R_* = 0.30], and total GRCS score [*χ*^2^ (2) = 65.55; *p* < 0.001; *ε^2^_R_* = 0.34]. Group means, standard deviations, and *post hoc* comparisons are shown in Tables 2, 4.

**Table 4:** Post hoc comparisons after controlling the overlap between the groups.

	Comparisons		*U*	*p*	*r*
Illusion of control	Problematic gamblers	Loot boxers	1,068	0.109	0.14
		Non-problematic gamblers	809	0.152	0.15
	Loot boxers	Non-problematic gamblers^*^	1850.5	< 0.001^*^	0.39
Predictive control	Problematic gamblers	Loot boxers	1,189	0.394	0.08
		Non-problematic gamblers^*^	659.5	0.010^*^	0.26
	Loot boxers	Non-problematic gamblers^*^	1,608	< 0.001^*^	0.44
Interpretative biases	Problematic gamblers	Loot boxers	1088.5	0.144	0.13
		Non-problematic gamblers^*^	338	< 0.001^*^	0.53
	Loot boxers	Non-problematic gamblers^*^	1,319	< 0.001^*^	0.52
Gambling-related expectancies	Problematic gamblers	Loot boxers	1271.5	0.723	0.03
		Non-problematic gamblers^*^	327.5	< 0.001^*^	0.54
	Loot boxers	Non-problematic gamblers^*^	559.5	< 0.001^*^	0.72
Perceived inability to stop gambling	Problematic gamblers	Loot boxers^*^	703	< 0.001^*^	0.35
		Non-problematic gamblers^*^	234	< 0.001^*^	0.65
	Loot boxers	Non-problematic gamblers^*^	1658.5	< 0.001^*^	0.46
Total GRCS score	Problematic gamblers	Loot boxers	1192.5	0.407	0.08
		Non-problematic gamblers^*^	302	< 0.001^*^	0.54
	Loot boxers	Non-problematic gamblers^*^	989.5	< 0.001^*^	0.60

* Statistical significance *p* ≤ 0.05.

Participants classified as gamblers completed the GRCS, while participants classified as loot box users completed the GRCS-LB.

The results of the *post hoc* tests indicated that problematic gamblers and loot boxers shared numerous cognitive biases (illusion of control, predictive control, interpretative biases, and gambling-related expectancies). Problematic gamblers and loot boxers scored higher than the non-problematic gamblers group in the cognitive biases assessed, except for the illusion of control, where no differences were found between problematic gamblers and the non-problematic gamblers group.

## 4 Discussion

This study aimed to explore the presence of shared cognitive biases between problematic gamblers, LB purchasers and free-LB openers, and to compare them with a non-problematic gamblers group. Our main finding suggests that problematic gamblers, LB purchasers and free-LB openers share several cognitive biases and that these biases are higher among these groups than in the non-problematic gamblers group, even when the overlap between the groups is controlled. Additionally, shared cognitive biases were observed between individuals who purchase LBs and those who open them for free, but with a different intensity (stronger biases were observed in purchasers).

Our results did not reveal differences between problematic gamblers, LB purchasers and free-LB openers in illusion of control or predictive control. The presence of the illusion of control suggests that, as happens in gambling, LBs might induce a greater perception of expertise and control over the items video game players are about to obtain from the boxes, even though these items are uncontrollable (Griffiths, 1993). Certain mechanisms seem to foster the emergence of this bias in gambling, such as the presence of features that lead players to falsely perceive they are playing games of skill instead of gambling (e.g., competition, choice, familiarity, and involvement; Langer, 1975). These elements may also be present in certain LBs. For example, in Hearthstone, players can choose between card packs of different colours. Although the probability of obtaining the most valuable items (i.e., legendary cards) remains unchanged, the possibility of choosing may engender beliefs about having influence on the outcome of these random events.

Regarding predictive control, our results suggest that LB purchasers and free-LB openers, like gamblers, believe in their ability to make accurate predictions of the outcomes. These predictions are based on salient cues (e.g., in LB, these cues may be represented by differential features of the loot box such as being brighter, more intense colours, etc.), probability errors and the player’s history of wins/losses (Raylu and Oei, 2004). The latter is related to the gambler’s fallacy, which consist in perceiving an outcome as being more probable if it has not occurred after a series of attempts (Lambos and Delfabbro, 2007) or if it has occurred on several occasions (Bersabé, 1995). An example of this phenomenon in LBs occurs in the FIFA video game: after opening a certain number of packs, if a player has not obtained the expected item (e.g., a player with a score higher than 84 in Ultimate Team), they might think they are about to achieve it. However, if they do obtain it, these can lead to the believe that they are ‘on a roll’ and that he could obtain another item with similar characteristics. The belief that they predicted the outcome could encourage them to keep opening or purchasing LBs, as happens in gambling, particularly if it follows a near-miss (Billieux et al., 2012).

No differences were found between problematic gamblers and LB purchasers in interpretative biases, gambling-related expectancies or in the total GRCS score, but there were differences between problematic gamblers and LB openers. The similarity in interpretive biases between LB purchasers and problematic gamblers suggests that video gamers may continuously re-evaluate their experiences in purchasing LBs, mostly recalling the wins and minimising their losses (Fortune and Goodie, 2012). Gamers could be attributing gains to internal variables (e.g., skill) and losses to external factors (e.g., luck), which may reduce subjective discomfort about not winning. The appearance of this bias in LB purchasers may encourage the maintenance of purchasing behaviour, in the same vein as gambling (Estévez et al., 2021).

Similarities between problematic gamblers and LB purchasers in gambling-related expectancies suggest that both could share beliefs regarding the benefits of gambling for or purchasing LBs. These benefits include socialising, coping with negative emotions, excitement-seeking (Ruiz de Lara and Perales, 2020), material gains and positive self-evaluation (Wickwire et al., 2010). An example of socialising in LBs could be one player holding a character in NBA 2K MyTEAM that is difficult to obtain (e.g., LeBron James) and which their friends do not have but with whom they want to play. Positive expectancies could maintain purchase behaviour, as Gillespie et al. (2007) described for gambling.

Contrary to what was expected, differences were observed between problematic gamblers, LB purchasers and LB openers in their perceived inability to stop gambling, with problematic gamblers reporting a higher impairment of control. Although LB purchasers and openers experienced negative reactions associated with the use of LBs, such as distress, guilt and loss of control (Sanmartín et al., 2021), these feelings do not appear to be sufficiently intense to affect their ability to stop purchasing LBs.

Differences were observed between problematic gamblers, LB purchasers and free-LB openers compared to the non-problematic gamblers group in all cognitive biases and in the total GRCS score, with the non-problematic gamblers group scoring the lowest. In LBs, these results are consistent with Brooks and Clark (2019), who reported a positive association between risky LB use and GRCS. Similarly, our results are in line with findings from the literature on gambling, where problematic gamblers present higher scores than non-problematic gamblers on these biases (Tang and Wu, 2012; Ciccarelli et al., 2017).

No differences were found between LB purchasers and LB openers in any cognitive bias examined or in the total score of the GRCS, although the purchasers obtained higher scores. This finding is relevant since it suggests that LB purchasers and LB openers share many common biases, even though the literature has focused on purchasers. Although they differ in the expenditure to obtain the items (which could be indicative of greater engagement), openers are also subject to the same mechanics of chance. Consequently, future studies should include LB openers to determine whether there are similarities between purchasers and openers in other variables, as in this study.

To strengthen the conclusions of our results, the overlap between the groups composing the sample was examined. After this process, our results remained similar. Problematic gamblers and loot boxers obtained statistically similar scores in several cognitive biases, including illusion of control, predictive control, interpretative biases, and gambling-related expectancies; and scored higher than the non-problematic gamblers group in all cognitive biases except for the illusion of control, where no differences were found between problematic gamblers and non-problematic gamblers. A possible explanation for this difference may be associated with the effect of psychotherapy on cognitive biases. Previous studies have reported that time spent in psychological treatment is associated with a decrease in cognitive biases (Stojnić et al., 2019). Therefore, excluding problematic gamblers who used loot boxes (who were younger and, consequently, received less therapy compared to traditional gamblers) from the sample reduced the average score for cognitive biases. Thus, the differences between the problematic gamblers and the non-problematic gamblers group diminished.

Our results suggest that the use of LBs may be explained along a continuum in which the different groups are distributed according to their similarity to problematic or pathological gamblers. Thus, the non-problematic gamblers group (i.e., individuals who had never opened or purchased LBs, but who had gambled in the past 12 months) would be at the lower end of this continuum, followed by LB openers and then LB purchasers. Finally, problematic or pathological gamblers would be located at the upper end of the continuum.

This proposal could explain the gradual approximation of loot boxers to gambling. In certain video games, LBs are presented as free articles and are easy to obtain, mainly by achievements (e.g., obtaining level 5). For the player, obtaining items within the game may be a way to speed up their progress, increase their competitiveness or change their appearance. Regardless of a player’s motivation to open LBs, they could act as an entry to initiate purchase behaviour, since free openings are not always sufficient to obtain valuable items (since the probability of obtaining them is low). Players who decide to purchase LBs are more likely to obtain the expected items than with free-openings because they are allowed a higher number of attempts or because the probabilities of obtaining the items increase (e.g., the more expensive the LBs, the greater the chances of obtaining the items). In either case, purchasing behaviour is likely reinforced and maintained when the player obtains valuable items. Finally, normalisation and the potential instrumentalisation of gambling as a way to obtain the desired items may be an entry to other gambling forms (e.g., slot machines), which may explain the similarities between problem gamblers and LB purchasers reported in previous studies.

Despite the potential interest of our results in the debate about the relationship between LBs and gambling, this study has certain limitations. First, although the sample size may seem small (especially regarding problematic gamblers), it is important to keep in mind the specific nature of this sample and the difficulty of accessing the participants, along with the relevant contribution of this sample to the debate about LBs as a form of gambling. Second, although LB openers and purchasers were distinguished in our study, the GRCS-LB combines both behaviours within its items (i.e., “*I’m not strong enough to stop opening or buying loot boxes*”). As a result, the questionnaire does not allow for a clear separation between openers and purchasers. Future research should aim to differentiate these behaviours to better determine any potential differences between them. Additionally, to the best of our knowledge, very few studies to date have explored cognitive biases, thus making it difficult to compare our data. Consequently, the theoretical background of this article was based on the gambling literature. Finally, is important to underscore the inherent limitations of cross-sectional studies and online data collection. It would be interesting to conduct longitudinal studies to further explore the relationship between LBs and gambling, and to analyse whether a behavioural migration occurs. Future studies should further examine how cognitive biases influence the opening and purchase of LBs.

In summary, our results suggest the existence of shared cognitive biases between problematic gamblers and loot boxers that may be associated with the convergence that previous studies have reported. Although further studies are necessary, results such as those presented in this paper aim to contribute scientific evidence to the ongoing debate on whether LBs are a form of gambling or if they constitute a separate phenomenon.

## Data Availability

The raw data supporting the conclusions of this article will be made available by the authors, without undue reservation.
