# Supplementary material for: Do problematic gamblers and loot boxers share similar fallacies of thought? A comparative analysis of cognitive biases
Source: Front Psychol. 2024 Nov 26;15:1430926. doi: 10.3389/fpsyg.2024.1430926 (PMC11628250; doi:10.3389/fpsyg.2024.1430926)
Supplement: Supplementary file 1 [file Table_1.DOCX]

Supplementary Material

# Supplementary Data

*Adaptation of the Gambling-Related Cognitions Scale (GRCS) to Loot Box use*

Please indicate the extent to which you agree with the value expressed in each statement, where

1 = strongly disagree; 2 = moderately disagree; 3 = mildly disagree; 4 = neither agree nor disagree; 5 = mildly agree; 6 = moderately agree; 7 = strongly agree

1. Opening free loot boxes or buying them makes me happier.
2. I can’t function without opening or buying loot boxes.
3. Praying helps me get the items I want.
4. Not getting the items I want are bound to be followed by a run of getting the items I want.
5. Relating getting the items I want to my skill and ability makes me continue opening or buying loot boxes.
6. Opening or buying loot boxes makes the video game seem better.
7. It is difficult to stop opening or buying loot boxes as I am so out of control.
8. Specific numbers and colours can help increase my chances of getting the items I want.
9. Not getting a series of items I want will provide me with a learning experience that will help me get them later.
10. Relating not getting the items I want to bad luck and bad circumstances makes me continue opening or buying loot boxes.
11. Opening or buying loot boxes makes the future brighter.
12. My desire to open or buy loot boxes is so overpowering.
13. I collect specific objects that help increase my chances of getting the items I want.
14. Once I get an item I want, I will definitely continue to get them.
15. Relating not getting the items I want to probability makes me continue opening or buying loot boxes.
16. Opening or buying loot boxes helps reduce tension and stress.
17. I’m not strong enough to stop opening or buying loot boxes.
18. I have specific rituals and behaviours that increase my chances of getting the items I want.
19. There are times that I feel lucky and open or buy loot boxes those times only.
20. Remembering the items I got last time makes me continue opening or buying loot boxes.
21. I will never be able to stop opening or buying loot boxes.
22. I have some control over predicting when I will get the items I want.
23. If I keep changing the loot boxes I open or buy regularly, I have less chance of getting the item I want than if I keep opening and buying the same loot boxes every time.
